# Sex differences in cooperativeness—An experiment with Buryats in Southern Siberia

**DOI:** 10.1371/journal.pone.0239129

**Published:** 2020-09-11

**Authors:** Victoria V. Rostovtseva, Franz J. Weissing, Anna A. Mezentseva, Marina L. Butovskaya

**Affiliations:** 1 Department of Cross-Cultural Psychology and Human Ethology, Institute of Ethnology and Anthropology, Russian Academy of Sciences, Moscow, Russia; 2 Groningen Institute for Evolutionary Life Sciences, University of Groningen, Groningen, The Netherlands; 3 Netherlands Institute for Advanced Study in the Humanities and Social Sciences, Amsterdam, The Netherlands; 4 International Centre of Anthropology, National Research University Higher School of Economics, Moscow, Russia; Middlesex University, UNITED KINGDOM

## Abstract

We report on an experimental study that was set up to reveal differences in the tendencies of men and women to cooperate in same-sex interactions. Former studies on this subject were mostly conducted in industrialized modern societies. In contrast, we tested the cooperation tendency among Buryats, a people from Southern Siberia of Mongolian origin. All subjects participated in (1) one iterated Public Goods Game in a group of four individuals of the same sex and (2) four one-shot Prisoner’s Dilemma games with different partners of the same sex. The interactions were in a face-to-face setting, but any intentional communication during the experiments was prohibited. We found that Buryat men were more cooperative than Buryat women in both types of same-sex interactions. In particular, the fraction of men employing a strategy of unconditional cooperation in the iterated Public Goods Game was much higher (36%) than the fraction of unconditional cooperators among women (21%). In general, the behavior of men was less context dependent than the behavior of women. In both sexes, individuals who were more cooperative in one type of game tended to be more cooperative in the other type of game. Although direct communication was prohibited, the interaction partners in the Prisoner’s Dilemma games employed the same strategy much more frequently than expected by chance. We conclude that, even among strangers, the exchange of subtle signals is sufficient to coordinate strategic decisions.

## Introduction

We report on an experimental study that was set up to reveal differences in the tendencies of men and women to cooperate in same-sex interactions. Sex differences in cooperative behavior have been experimentally studied for more than 50 years. Yet, only few of these studies focus on interactions with individuals of the same sex. The most extensive survey of the literature on the subject [1] reviews only 10 studies considering same-sex cooperation in the context of public goods provisioning. The results of these studies were inconsistent: four studies report that women tend to be more cooperative in same-sex situations [2–5]; one study found men to be more cooperative [6]; and five could not detect significant sex differences [7–11]. Moreover, the scope of these studies may be limited, because nine of them were conducted in Western societies (USA, Canada), and two in Japan (with one comparing Canadian and Japanese subjects [5]). It is well-known [12–15] that there are substantial cultural differences in cooperation, making it important to conduct experiments with non-WEIRD participants (WEIRD: Western, educated, industrialized, rich and democratic [16]). The recent study by Dorrough and Glöckner [17], used a continuous version of the Prisoner’s Dilemma game (actually a two-person Public Goods Game) to investigate sex differences in cooperation in a diverse set of countries (Chile, Venezuela, Mexico, USA, England, Germany, Austria, Israel, Russia, India, Australia, and Japan). Overall, men tended to cooperate more than women, but there were substantial differences across countries: almost no sex differences were observed in Germany, USA, and Venezuela; sex differences were very pronounced in Japan, with men being more cooperative; and, in contrast to all other countries, women tended to be more cooperative than men in Russia. To our knowledge, results on cultural differences in the behavior of men and women in a same-sex group setting have been reported only in one study [5]. Therefore, to expand the scope of the studies to a non-WEIRD society, we conducted such experiments with members from the Buryat population.

Buryats are a people of Southern Siberia of Mongolian origin. They are traditionally nomadic pastoralists [18, 19], mostly living in Buryatia (the Baikal Lake area of the Russian Federation) [20–22]. Less than a century ago these people were typical representatives of a patriarchal, patrilocal society with a traditional pastoral economy [19]. Their exclusively-male activities involved sophisticated warfare practices and collective hunting in large groups of 300–1,000 men [23–25]. The role of women was mainly defined in terms of child care and domestic activities [26–28]. Traditionally, families were strongly patriarchal and members were related on the paternal side. Families were composed of several married couples and their offspring, and a common household could consist of several dozen members [27, 28]. By the beginning of the 20^th^ century, these large patriarchal families had disintegrated substantially, giving small families a more prominent role [29]. Nevertheless, the general tradition of large patriarchal families is still maintained in the Buryat culture [27, 28, 30–33]. In various ways, modern Buryats differ from Russians living in the same region; for example, they tend to have more children (4–6 children is the norm for modern Buryats, whereas Russians hardly reach the level of three children in a family) and the ‘patriarch’ still has a powerful role in most Butyat families [34]. Although many Buryats have adopted an urban life-style [22], there is a large body of evidence within social anthropology and social psychology that asserts that Buryats still have highly traditionally oriented mentalities regarding cultural norms [28, 32, 35–38]. Some scholars even apply the concept of “ethnic encapsulation” to the ethnic identity of modern Buryats [39]. The traditional religion of Buryats is Shamanism [40–42]. The major religious denomination in Buryatia is Buddhism [43], but the majority of Buryats relate to it only nominally [44].

Differences in cooperativeness can be investigated in a multitude of ways. Experimental studies are often based on economic games, such as the Dictator Game, the Ultimatum Game, the Trust Game, and many others [45, 46]. These games focus on different aspects of cooperation (e.g. generosity, trust, risk-taking, division of labor, etc.). As in the majority of earlier experiments (e.g. the experiments in [1] and [17]), our study is based on the Public Goods Game (PGG) [47, 48] and the Prisoner’s Dilemma game (PD) [49, 50]. Our main focus is on the PGG, which allows researchers to study cooperation in a group context. In this game, each individual in a group of, say, four members is asked to invest funds into a common pool from which all group members can profit. The sum of all investments is doubled and distributed over all group members, irrespective of how much they contributed to the common pool. Such a PGG is the prototype example of a social dilemma: because the content of the common pool is doubled, the profit of the group (and all individual group members) would be maximized if everybody invested as much as possible. Yet, from an individual perspective, it is rational to not invest at all, since each invested Dollar yields less than one Dollar in return (the Dollar is first doubled, but subsequently the two Dollars are distributed over the four group members, yielding half a Dollar per individual). In other words, individual-level interests may prevent the optimal group-level outcome. A PGG, in particular if it is iterated, has a multitude of strategies that can be difficult to analyze. Therefore, we also let our subjects play several Prisoner’s Dilemma (PD) games, which have a much simpler structure. The two players of a (single-shot) PD game have only two options: cooperate or defect. The payoff configuration of the game is such that mutual cooperation yields a higher payoff for both players than mutual defection. Yet, it is rational not to cooperate, because regardless of the other player’s actions defection yields a higher individual outcome than cooperation. This property makes the PD game the prototype of a social dilemma in a dyadic (= two-person) context.

The participants of our experiment first played an iterated Public Goods Game in a fixed group of four same-sex participants. The iterated PGG has a rich strategic structure, allowing observers to analyze in considerable detail the build-up and/or decay of cooperation. To obtain a relatively simple measure of cooperation tendency, we also let the participants play four one-shot Prisoner’s Dilemma games with different same-sex interaction partners. We are aware that carry-over effects are unavoidable when subjects play cooperation games in the same sequence. We chose the sequence “PGG first–PD games later” because we want to avoid such carry-over effects for the PGG, which was our main focus. To keep the participants at ease, we used a face-to-face design, where interaction partners could see each other, without being allowed to communicate directly. Such a setting is closer to “real life” but implies that the participants can exchange (consciously or unconsciously) subtle signals that are not noticed by the experimenters. The evidence suggests [51] that the level of cooperation is generally higher in face-to-face settings, and our results also reveal that the participants of our experiment coordinated their choice of behavior (see the Discussion). Therefore, a comparison of our results with those of other studies (which were typically conducted in an anonymous setting) should be done with caution. Yet, for our research question (Do Buryat men and women differ in their cooperation strategies when interacting with same-sex partners?) such design questions are of minor importance, since male and female participants were exposed to the same treatment.

## Material and methods

Our experiments were conducted with 208 Buryats (104 men, 104 women), students in Ulan-Ude (the capital of Buryatia) of different specialties (natural and social sciences, economics, arts). Many of the participants had come from rural areas to study in the capital. All were bilingual, speaking both the Russian and the Buryat languages. To minimize possible age effects, we restricted the age interval of our subject pool to 17–25 years (mean age 20 ± 2 y). For this reason, two participants were excluded from the analysis. Twelve other individuals (six pairs) were excluded, because they were acquaintances. Our final sample, therefore, consisted of 194 individuals (97 men, 97 women). Within the given age range, statistical analysis did not reveal any age effects on the experimental parameters.

All subjects participated in five cooperation games with interaction partners of the same sex: (1) one iterated Public Goods Game (iPGG) with a group of four interaction partners; and (2) four dyadic one-shot Prisoner’s Dilemma games with different partners. The study was conducted in two parts: the first two weeks only male participants were recruited, and the next two weeks only females. Every experimental session involved eight subjects, who were strangers to each other. Upon arrival, the eight participants of a session were placed in two separate rooms (four subjects per room) for the iPGG group interactions. After the iPGG was completed, the participants of the two groups were assorted in one room for the PD interactions. To this end, each participant played one PD game with each of the four members of the other iPGG group. This way each participant interacted with seven different subjects.

The experiment was held in the Russian language, which is native both for the experimenters and participants of the study. Prior to the experiment, the subjects were informed that during the interactions they would earn tokens that would be exchanged for real money at the end of the experiment. The exchange rate was not announced. This was done due to possible differences in income levels between participants, which could cause a kind of inequality in financial interests during the game. However, the participants were informed that the average overall payoff would be around 1000 Rubles (20 USD), the realized payoff being largely dependent on individual performance. All subjects signed informed consents prior to the experiment. Since all minors were older than 16 years of age, additional consent from parents was not required. The study was approved by the scientific board of the Institute of Ethnology and Anthropology of the Russian Academy of Sciences.

The iterated Public Goods Game was played in groups of four subjects of the same sex, who interacted with each other in three identical rounds. Groups were formed so that the group members were stranger to each other. During the game, the members of a group were seated at a table facing each other. Any intentional communication (negotiations, signs, gestures, or intentional facial signaling) between participants was not allowed during the entire course of the interaction. To ensure that no communication happened, the experimenter was present in the room for the full duration of the experiment. Moreover, the experiment was video-recorded. Post-hoc observation did not reveal any obvious signs of intentional communication between participants during experimental interactions. Before the game started, the rules were explained in detail to the participants. In each round, each participant was given 20 initial tokens. Then, each participant had to decide (privately and without negotiations or communication) how many tokens (from 0 to 20) to invest in a common “project”. Participants had to write the amount on a personal sheet, which was observable only to him/her-self and the experimenter. All participants were informed and reassured, that none of the group-partners would get to know the amounts invested by others during the entire experiment. Tokens not invested were kept by the participant. When all group members had made their investment decisions, the invested tokens were doubled and distributed equally over all four group members. If the doubled amount was not dividable by four, the outcomes were reported precise to decimals. The number of retuned tokens was announced to all group members, but this number does not provide any information on individual investments.

After the iPGG was completed, participants were invited to a different room, where pairs were formed for dyadic PD interactions. Each participant was involved in four one-shot interactions with different partners of the participant’s sex. In each of these interactions, both partners had to choose one of two options: to cooperate, or to defect. Before, they had been informed about the possible outcomes: if both partners cooperated, each got five tokens; if both defected, each got two tokens; if one cooperated and one defected, the former got one token, and the latter eight tokens. The experimental procedure was as follows: In each interaction, the subjects were seated facing each other. As in the iPGG, they were asked to write their decision (“cooperate” or “defect”) on a personal sheet; this decision was visible to the experimenter but hidden to the interaction partner. When both participants had made their decisions, the experimenter wrote down the number of tokens earned on the personal sheets of the participants (not visible to the other). Hence all decisions and result announcements were made privately and in silence, implying that none of the participants could obtain information on their partner’s previous decisions. After an interaction was completed, the game partners were reshuffled before new interaction proceeded in the same way. During the PD sessions, two experimenters were present in the room for the full duration of the experiment to ensure that there was no communication between participants.

The number of rounds (three) of the iPGG and the number of repetitions (four) of the PD game were mainly dictated by logistic and financial constraints: at least three rounds of the iPGG are required to unravel strategic patterns, and four repetitions of the PD interaction (each with a new stranger partner) could easily be implemented in our spatial setting (eight people in one room, see above).

Since the PGG was played in the iterated manner, it allowed getting an insight into strategic behavior, which was defined based on the variation of individual investments across all subsequent interactions. Analysis of investments allowed distinguishing certain strategic types, such as conditional/unconditional cooperation, self-oriented behavior, occasional free-riding, or cheating. Unconditional strategies imply consistency of investments across all interactions, regardless of the outcomes of the previous rounds. This means that participants who applied unconditional strategies stuck to the same level of investments (whether high or low) and did not vary it according to the outcomes of the previous interactions. Conditional strategies, in contrast, imply flexible behavior and adjustment of investments according to the outcomes of the previous rounds. Generally, such an approach is based on the concept of social responsiveness vs. behavioral consistency, which is well-known in evolutionary biology [52, 53]. The strategy method strives to obtain insights into how individuals arrived at their decisions and whether individuals differ in their decision-making. Since the number of strategies is astronomic even in simple repeated games (6 * 10^7 in the iPGG of our experiment), strategies need to be classified into plausible “rules of thumb”. After defining strategic types, further analysis can be conducted. The exact investment thresholds for each type of strategies were set up based on variation in individual investments in this particular study. Absolute classification thresholds, which could fit different studies universally, are not existent and cannot be applied, since absolute levels of investments may vary across contexts and across populations. Generally, we distinguished five strategies based on variation in individual decisions across three rounds of the iPGG. Participants, who largely varied investments across rounds were classified as “conditional cooperators” (e.g. 7 tokens [1st round], 10 tokens [2nd round], 15 tokens [3rd round] (“conditional, starting low”); or 20 tokens [1st round], 10 tokens [2nd round], 5 tokens [3rd round] (“conditional, starting high”)); those, who invested 0 at least once were called “occasional free-riders” (see below). Both of these strategies are responsive. If a participant varied investments only in uppercase interval of investments (≥75% of own funds), he/she was classified as an “unconditional cooperator” (e.g. 20 tokens [1st round], 15 tokens [2nd round], 17 tokens [3rd round]; or 20 tokens [1st round], 15 tokens [2nd round], 20 tokens [3rd round]). If a participant varied investments only in lowercase interval of investments (<50% of own funds), he/she was classified as “unconditionally self-oriented” (e.g. 4 tokens [1st round], 6 tokens [2nd round], 8 tokens [3rd round]; or 8 tokens [1st round], 2 tokens [2nd round], 9 tokens [3rd round]. The variation of investments for unconditional cooperators and self-oriented individuals took place only within the upper- or lower-case intervals of absolute range of investments. This did not allow classifying them as “conditional cooperators,” because there was a considerable fraction of participants who varied investments widely within the whole range of possible investment amounts. Only five cases did not fit our scheme (mainly those who always invested between 50% and 75%). They were excluded from the analysis.

## Results

### Iterated Public Goods Game

**Fig 1:**
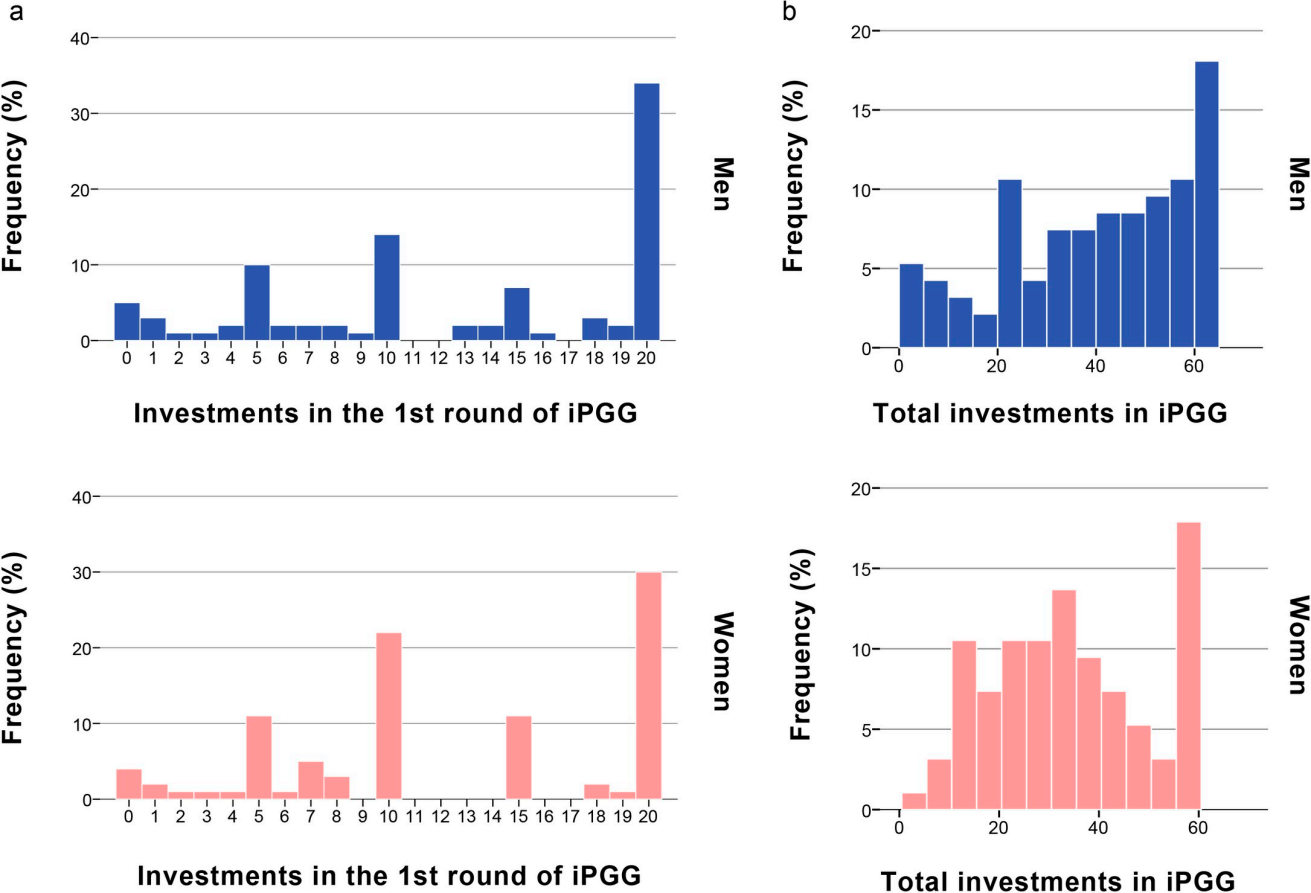
**The frequency distribution of first (a) and total (b) investments in the iterated Public Goods Game among male and female participants.** (a) Distributions of the first investments of men and women do not differ significantly: Mann-Whitney U-test: N = 189, U = 4297.5, p = 0.648; (b) distributions of the total investments of men and women do not differ significantly: Mann-Whitney U-test: N = 189, U = 3910.5, p = 0.139.

Fig 1A shows the individual investments of men and women in the first round of the iPGG. Fig 1B displays distributions of total investments. The investment in the first round may be viewed as a summary of statistics for the general cooperation tendency of the subjects and their overall expectations of the cooperativeness of others. Moreover, this initial behavior is not yet affected by the peculiarities of the group. The distribution of initial investments does not seem to differ much between men and women. This is confirmed by a Mann-Whitney U-test that does not detect any significant differences (N = 189, U = 4297.5, p = 0.648).

The interpretation of the initial investment in the first round as an indicator of the general cooperation tendency is not fully convincing. The participants of our experiment were aware from the start that the PGG would be played repeatedly. Therefore, we cannot exclude the possibility that participants had longer-term strategic considerations when making their first move. For example, a high initial investment could indicate either a high general cooperation tendency or the attempt to establish trust in other group members that could later be exploited in the subsequent rounds. However, the distributions of total investments differ between men and women (Fig 1B). Therefore, a more sophisticated analysis of the iPGG is required. Such an analysis should include the investments made in all three rounds, and place these investment decisions in a strategic perspective.

As a first step, we used an intra-class correlation analysis to investigate whether men or women are more consistent in their investment decisions across the three rounds of the iPGG. It turned out the Intra-class Correlation Coefficient (ICC) was 0.584 in men (N = 94) and 0.401 in women (N = 95). The fact that the ICC of men is substantially larger than the ICC of women could indicate that women are more responsive in their investment decisions, while men are more consistent.

To obtain deeper insights into the male and female strategies (and their differences), we categorized the participants on the basis of their investment pattern into five broad categories:

- Unconditional Cooperator (UC): participants who in each of the three rounds invested ≥ 75% of their endowment (even if group investments had been low in previous rounds);
- Unconditionally Self-oriented (SO): participants who in each round invested <50% of their endowment (irrespective of the group investments in the previous rounds);
- Conditional, Starting High (CH): participants who invested ≥ 75% of their endowment in the first round and subsequently reduced their investment, reaching a level ≤50%;
- Conditional, Starting Low (CL): participants who invested ≤50% of their endowmentin the first round and subsequently increased their investment, reaching a level ≥ 75%;
- Occasional Free-rider (OF): participants who invested > 50% of their endowmentin one or two rounds and reduced their investment to (almost) zero in at least one round.

This categorization is, to a certain extent, *ad hoc*, but it reflects plausible behavioral patterns that are often observed in iterated Public Goods Games. It turned out that 189 of the 194 subjects of our study could be classified into one of these categories. The remaining five subjects (who all invested 50% to 75% of their endowment in all three rounds) are, for the rest of this section, excluded from the analysis.

**Fig 2:**
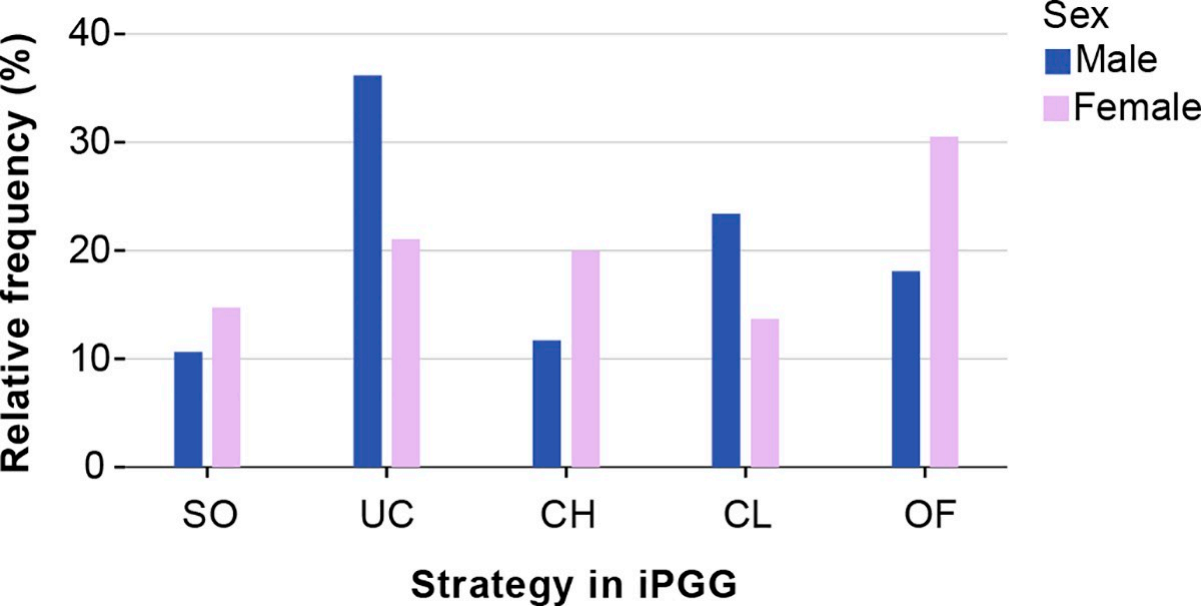
The frequency distribution of strategies in the iterated Public Goods Game in male and female participants. Self-oriented (SO): always invested < 50% of own funds into common pool; Unconditional cooperator (UC): always invested ≥ 75% of own funds, even if in previous rounds cooperation failed; Conditional, Starting High (CH): started with high investments and further declined (at least in one of subsequent interactions); Conditional, Starting Low (CL): entered interactions with low investments, but increased over the course of the game (at least in one of subsequent interactions); Occasional Free-rider (OF): invested 0 or suddenly crucially reduced investments amid high investments of other participants in the group.

Fig 2 displays the frequency distribution of these investment patterns and the differences between men and women in employing these strategies. A chi-squared test of independence reveals that the frequency distributions of strategies differ significantly between men and women (X^2^ = 12.003(4), p < 0.017). Men tended to be unconditionally cooperative more often (36%) than women (21%), and women tended to apply occasional free-riding more often (30%) than men (18%). Hence, our results indicate that, in comparison to women, Buryat men are more inclined to exhibit unconditional cooperation and less inclined to free-ride in same-sex interactions.

**Fig 3:**
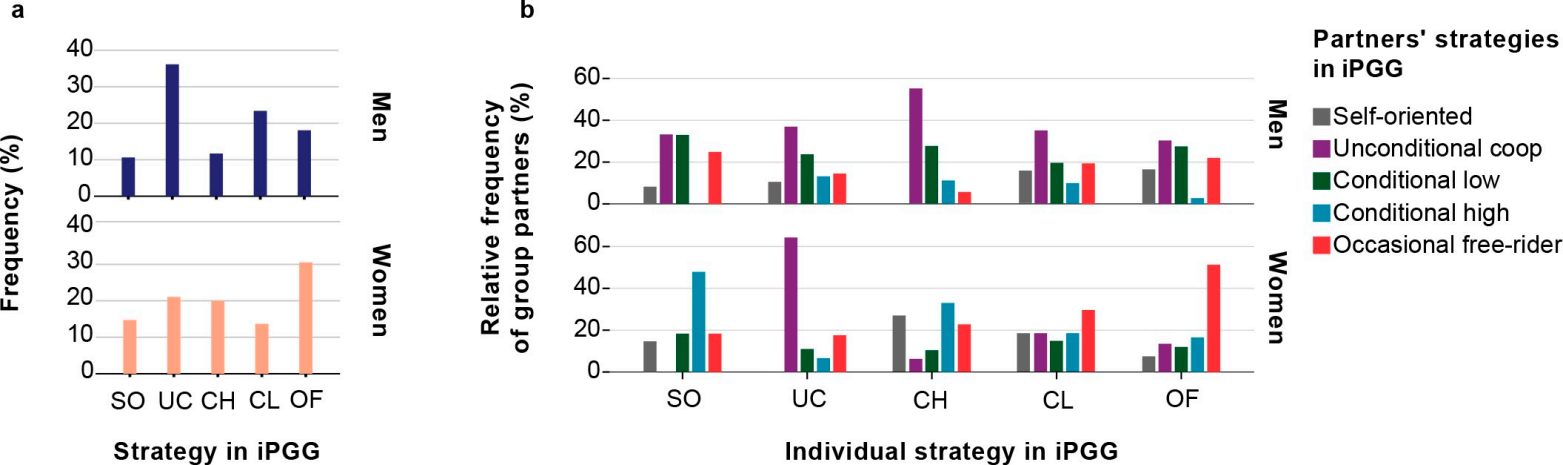
**The frequency distribution of strategies in the iterated Public Goods Game among (a) all men and all women and (b) among the partners of men and women that employed a specific strategy.** Strategies in the iPGG: Self-oriented (SO): always invested < 50% of own funds into common pool; Unconditional Cooperator (UC): always invested ≥ 75% of own funds, even if in previous rounds cooperation failed; Conditional, Starting High (CH): started with high investments and further declined (at least in one of subsequent interactions); Conditional, Starting Low (CL): entered interactions with low investments, but increased over the course of the game (at least in one of subsequent interactions); Occasional Free-rider (OF): invested 0 or suddenly crucially reduced investments amid high investments of other participants in the group.

According to the pay-off rules of the Public Goods Game, investing nothing (free-riding) always yields a higher short-term payoff than investing part of one’s budget (see Methods). Furthermore, in the finitely iterated Public Goods Game, free-riding is the only Nash equilibrium strategy. Yet, cooperation can be an efficient strategy if it induces other participants to invest in the public good. To investigate whether the observed strategies were used in a condition-dependent and strategic manner, we determined the frequency distribution for each of the strategies employed by the other group members. This way, we can address questions such as: is unconditional cooperation frequently accompanied by free-riding? Not all individuals could be included in the analysis, because groups had to be entirely removed if they contained one or more members who had been initially excluded due to different reasons. Fourteen participants were initially excluded from the analysis (see Methods), which caused exclusion of 14 groups, and four more individual strategies could not be identified according to our scheme, which caused exclusion of four more groups. This group-wise exclusion was necessary since participants from groups, in which information regarding behavior of each member was incomplete, could not be properly analyzed on the matter of behavior of their group-partners. The final overall data set for this analysis consisted of 139 subjects (68 males, 71 females). A chi-squared test for goodness-of-fit indicates that the N = 139 distribution does not differ significantly from the N = 189 distribution. Moreover, the small chi-squared value (X^2^ = 0.031) indicates that the difference between these distributions is negligible. The chi-squared test for goodness-of-fit that was applied to the male part of the sample did not reveal any significant differences between the strategy distributions of the partners for each of the five individual iPGG strategies and overall distribution of strategies in the male sample. As shown above (see also Fig 3A), unconditional cooperation was the most frequent strategy among men. Our additional analysis reveals (Fig 3B) that this holds true irrespective of the strategies of the partners. In other words, all strategies profited from male unconditional cooperators. In contrast, the strategy distribution of women was much more context dependent. When applied to the female part of the sample, the chi-squared test for goodness-of-fit reveals significant differences between the distributions of strategies employed against specific partner strategies and the overall distribution of strategies (Fig 3).

As demonstrated in Fig 3B, self-oriented women were more often accompanied by conditional cooperators, who started from high investments, but declined subsequently (X^2^ = 15.573(4), p = 0.004). Possibly, bad company was the cause of the decrease of initially high investments among conditional cooperators accompanied by self-oriented individuals. It also can be seen from the Fig 3B, that female unconditional cooperators were often accompanied by unconditional cooperators (X^2^ = 11.757(4), p = 0.019), and occasional free-riding also frequently occurred in the same groups (X^2^ = 10.639(4), p = 0.031). Since we have not applied any assortment on the basis of individual qualities, and subjects did not have the opportunity to choose group-partners, it is reasonable to assume that female participants somehow adjusted their behavior according to the characteristics of the social environment.

### Prisoner’s Dilemma games

**Fig 4:**
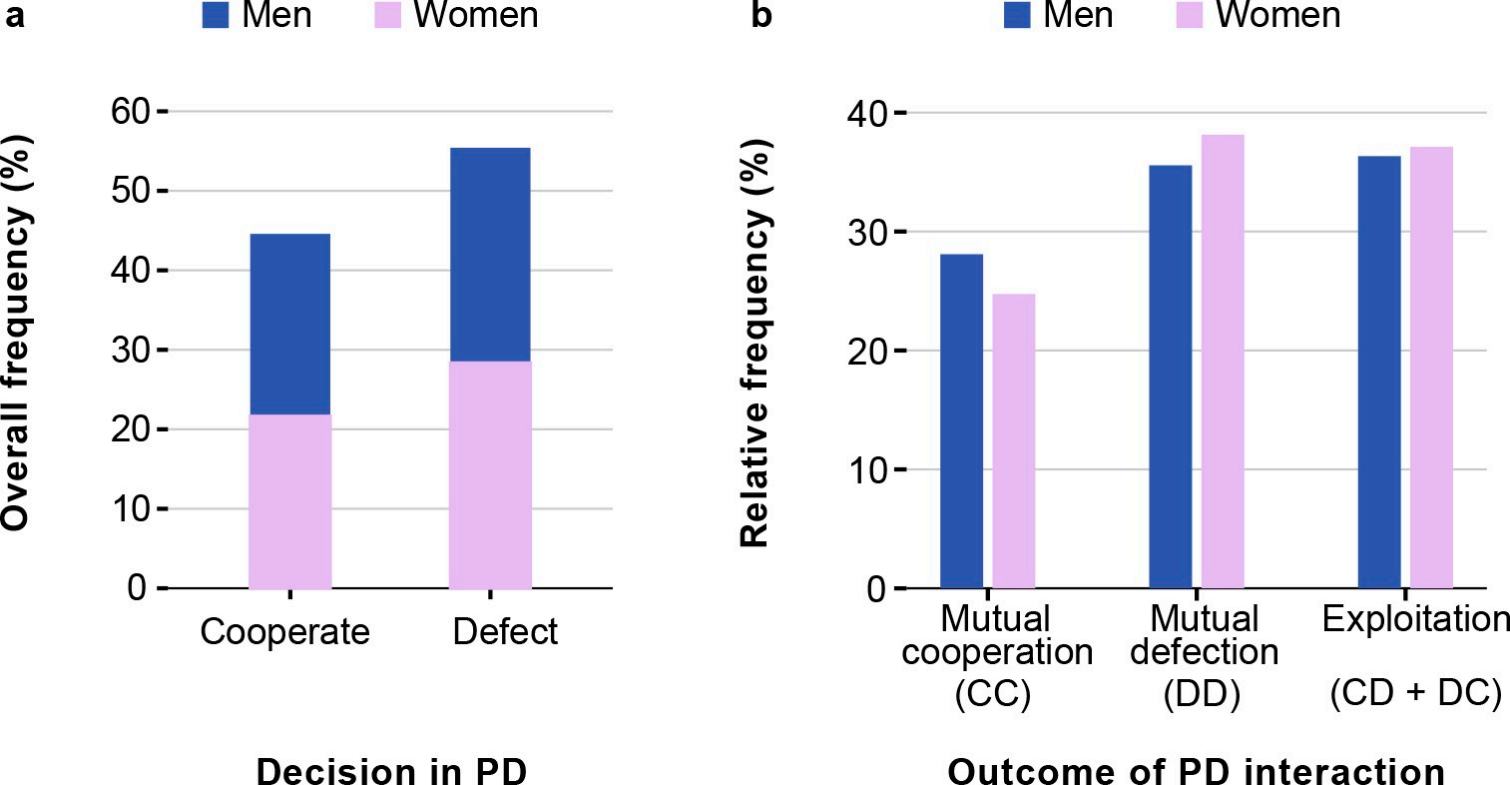
**The overall distribution of decisions (a) and the distribution of each interaction outcomes for men and women (b) in the Prisoner’s Dilemma games.** Classification of interactions: mutual cooperation (CC): both partners decided to cooperate; mutual defection (DD): both partners decided to defect; exploitation (CD or DC): partners have chosen different options.

In this section, we report on four rounds of the PD game, played by 97 male and 97 female participants. First, we estimated the general cooperativeness of our subjects playing the PD games. Fig 4A displays the overall frequencies of “cooperate” and “defect” decisions across all experimental interactions (N = 776 interactions). Overall, 55% of all decisions were to “defect” and 45% were to “cooperate,” meaning that generally defection occurred more often in PD than cooperation (a chi-square test for goodness-of-fit against equal distribution of decisions: X^2^ = 9.093, p = 0.003). However, no significant differences in the frequency of decisions “to cooperate” and “to defect” were observed in men (men cooperated in 45.9% of cases; p = 0.104), but at the same time women defected significantly more often (women cooperated in 43.3% of cases; p = 0.008). From the 194 participants under consideration, 76 (or 39%) were fully consistent in that they chose the same option in all four interactions with different partners. This means that all four decisions of those 76 subjects were either to cooperate, or to defect. Another 83 participants (43%) chose the same option in three of the four interactions. Only 35 participants (or 18%) were ambiguous in that they chose to cooperate in two of their interactions and to defect in the other two. A chi-squared test of independence indicated that men and women differed significantly in the consistency of their decisions (same decisions in 100, 75, or 50 percent of interactions) (X^2^ = 8.190(2), p = 0.017), with men performing in absolute agreement more often (50%) than women (29%).

To investigate how participants interacted in dyads, we classified the outcomes of the one-shot PD games as “mutual cooperation” (CC; both partners cooperated), “mutual defection” (DD; both partners defected), and “exploitation” (CD+DC; one partner cooperated while the other defected). If these outcomes reflected the *independent* choice of options by the two participants, one would expect the following relative frequencies of the three outcomes for men: CC 21% (= 0.459^2^), DD 29.25% (= 0.541^2^) and CD+DC 49.75% (= 2*0.459*0.541); and for women: CC 18.75% (= 0.433^2^), DD 32.25% (= 0.567^2^) and CD+DC 49% (= 2*0.433*0.567). A chi-square test for goodness-of-fit conducted for men (X^2^ = 28.613(2), p < 0.001) and women (X^2^ = 32.077(2), p < 0.001) clearly indicates that the decisions were *not* taken independently, despite the fact that all communication between partners was forbidden. In men and in women, the two interaction partners tended to choose the same option more often than would be expected by chance.

### Consistency across the two cooperation games

**Fig 5:**
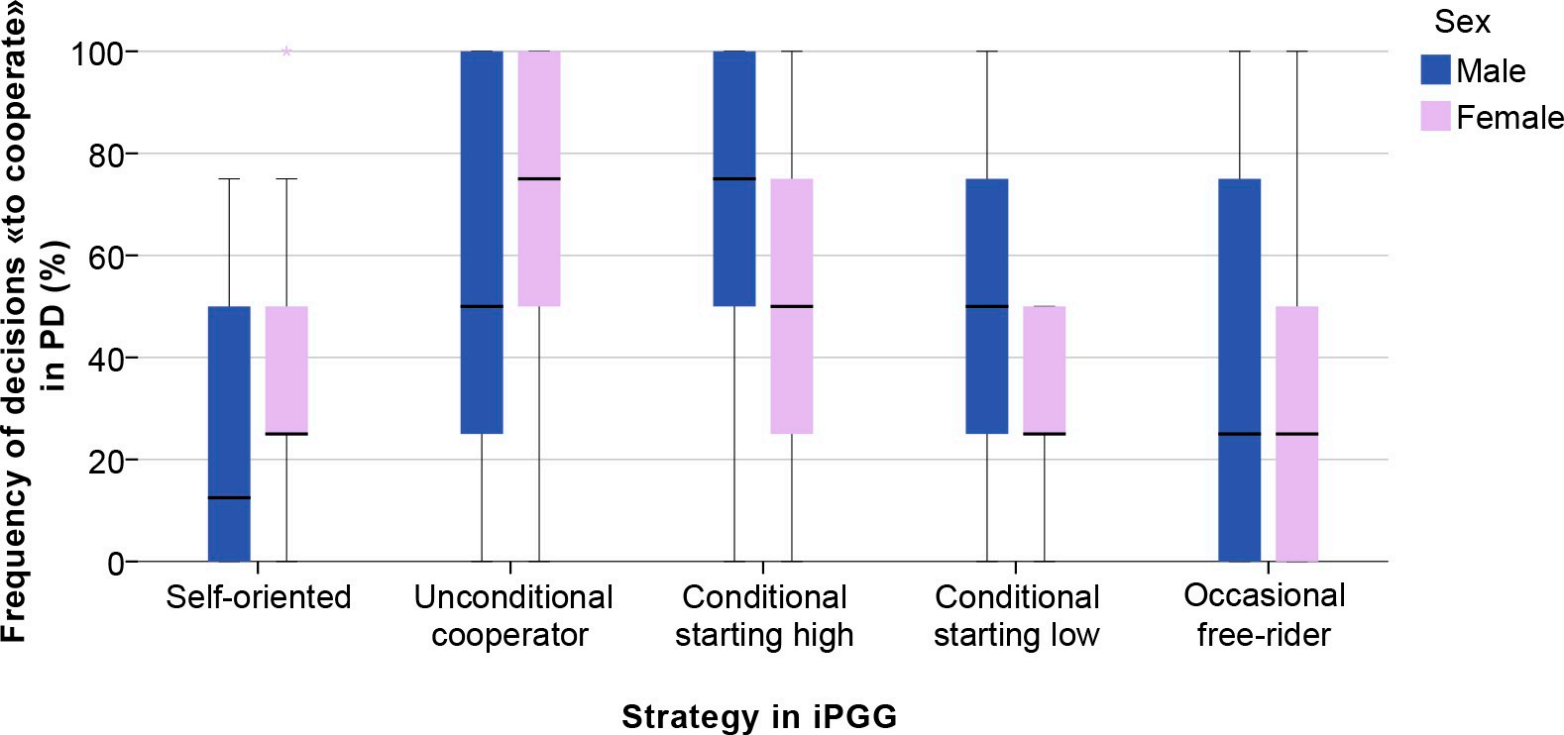
Correspondence of individual strategies in iterated Public Goods Game and cooperativeness in Prisoner’s Dilemma games for men and women. The dark line in the middle of the boxes is the median. The bottom of the box indicates the 25th percentile, the top of the box indicates the 75th percentile. The T-bars (whiskers) extend to 1.5 times the height of the box or, if no case has a value in that range, to the minimum or maximum values.

To investigate whether the same subjects behaved cooperatively in the iterated Public Goods Game and the Prisoner’s Dilemma games, we calculated a “PD cooperativeness” score for each individual (the percentage of decisions to cooperate over four interactions with different partners). Subsequently, we associated the PD cooperativeness with each of the five strategies observed in the iPGG (Fig 5). For men and women separately, we used a Tukey-Kramer test to investigate whether strategic classes in the iPGG were associated with a different level of PD cooperativeness. The Tukey-Kramer test could be employed because the distribution of PD cooperativeness values was approximately symmetric and with similar variance in each strategic class. For men, the Tukey-Kramer test did not detect significant differences in PD cooperativeness in relation to the iPGG strategy. For women, unconditional cooperators in the iPGG exhibited the highest PD cooperativeness; the level of PD cooperativeness in unconditional cooperators was significantly larger than that of women employing the “self-oriented,” the “conditional starting low,” and the “occasional free-rider” strategies.

## Discussion

A large body of literature from over 50 years of research in the field of sex differences in human cooperation shows that, generally (pooling both group and dyadic interactions) men tend to be marginally more cooperative and less context dependent than women [1]. However, upon closer examination of literature, it turns out that studies considering same-sex group cooperation come to contradicting conclusions; four studies report that women tend to be more cooperative in same-sex situations [2–5]; one study found men to be more cooperative [6]; and five could not detect significant sex differences [7–11]. Until today, these kinds of studies were mostly restricted to Western and highly industrialized societies (North America, Japan) characterized by certain cultural and socioeconomic conditions. This sampling bias substantially limits extrapolation of these findings to different geographic regions [16]. The recent work by Dorrough and Glöckner [17], measuring cooperativeness in continuous Prisoner’s Dilemma games across 12 different countries worldwide, has demonstrated that, although men generally tended to be more cooperative than women, results differed considerably between countries. Another important step which may bring us closer to understanding the mechanisms underlying same-sex cooperative behavior would be extension of the research area towards societies with different traditional socio-economic backgrounds.

The present study was conducted among Buryats of Southern Siberia—people with a nomadic pastoral economy and strong patriarchal traditions practiced during a long period of history up to the recent past. Our results have revealed that in both cooperation experiments, Buryat men showed a higher degree of cooperation in same-sex interactions than women. The behavior of men was also much less context dependent than the behavior of women. In the iterated Public Goods Game, a high fraction of men could be classified as unconditional cooperators (Figs 2 and 3A); moreover, even the men employing a condition-dependent strategy did not make their behavior dependent on the strategic choices of their partners (Fig 3B). In contrast, women were generally less cooperative in the iPGG, they more often applied occasional free-riding during the group interactions, and their actions were more context-dependent. They cooperated more in cooperative environments, and applied free-riding among free-riders. We can conclude with confidence that interactions in the iPGG generated *unconditional* cooperation in men, but not women. Therefore, our study demonstrates that among Buryats, men tend to be more cooperative in same-sex interactions, in comparison with women. However, in cross-cultural studies using similar experimental approaches, investigations of the universality of such presumably culturally-mediated tendencies need to be conducted. It is worth noting that one study conducted in Kenya with traditional patriarchal pastoralists (Orma), considering all-male group cooperation in the one-shot Public Goods Game, concluded that men in a given society were extremely cooperative in this particular game [54]. The study did not explore sex differences, but the author has compared cooperation rates to those obtained within American samples [47]. He arrived at the conclusion that in PGG Orma men were much more cooperative than Americans. This is the only study, to our knowledge, that considers all-male group cooperation in PGG in non-WEIRD society; however, its evidence is based only on 24 individuals.

In the iPGG and in the PD, Buryat men were also found to be considerably more consistent in their behavior (over interactions with different partners) compared to Buryat women, who demonstrated higher flexibility. Generally, a high degree of consistency in behavior may play an important role in social interactions with regard to needs for coordination, since consistent behavior of group members considerably enhances predictability of individual actions. Similar sex differences in consistency of cooperative behavior across numerous contexts were also found in one of our own experimental studies conducted in the Netherlands (manuscript in preparation). This behavioral phenomenon still needs to be explained.

A distinctive feature of our experiments with Buryats were the face-to-face interactions, where participants could see each other in a shared space and in real-time. Such experimental setting distinguishes our study from many other experiments, where strategic choices are made under strictly anonymous conditions. An anonymous experimental setting allows better control of interaction parameters, which increases internal validity. At the same time, such conditions are so artificial, that external validity of the results may be compromised. We are fully aware that even under condition of “no communication” information can be exchanged in a face-to-face setting. Our results indeed indicate that such exchange must have taken place. We have detected a surprising convergence of decisions to the mutual outcomes, which was especially noticeable among both sexes in the PD games (Fig 4B). Taking into consideration the lack of intentional communication during the experiment (negotiations, gestures, or intentional facial expressions were prohibited), and stranger terms of interactions, we are inclined to believe that some other forms of communication or nonverbal visual cues took place. There is evidence in the literature, suggesting that people can predict pro-social intentions based on just visual perception of potential partners [55–57]. Although appearance can be misleading in certain situations [58–60], *dynamic* nonverbal signals are considered informative and reliable. Numerous studies emphasize the importance of emotions and involuntary nonverbal expressions in the process of human cooperation [61–65]. Studies also show that emotional signals may modulate cooperativeness [66]. To our knowledge, our study is the first to demonstrate how subtle signaling may coordinate strategic behavior in a setting of face-to-face interactions. These results provide support for the existence and special role of nonverbal communication in the process of human cooperation.
